# An international comparison of gender differences in mental health among higher-education students during the first wave of the COVID-19 pandemic: a multilevel design

**DOI:** 10.1186/s13690-023-01211-2

**Published:** 2023-12-07

**Authors:** Sarah Van de Velde, Anneleen De Cuyper, Leen De Kort, Kimberly Jacobs, Nikoletta Somogyi, Robert Tholen, Nina Van Eekert, Veerle Buffel

**Affiliations:** 1 Centre for Population, Family, and Health, University of Antwerp, Kipdorp 61, Antwerp, 2000 Belgium; 2 Interface Demography, Vrije Universiteit Brussel, Pleinlaan 2, Brussels, 1050 Belgium

**Keywords:** Depression, Alcohol, Higher education, COVID-19, Multilevel

## Abstract

**Background:**

Mental health problems are a common phenomenon among higher-education students. How these mental health problems manifest themselves appears to differ between male and female students. While the latter group bears a greater risk of developing internalizing problems, with depression being particularly prevalent, these problems manifest themselves in male students mainly via externalizing disorders, with alcohol abuse being the most prevalent. Available cross-national research on students’ mental health during the COVID-19 pandemic, to date, mainly focused on the prevalence of depressive symptoms, thereby ignoring a possible gendered impact of the pandemic.

**Methods:**

The current study used the COVID-19 International Student Well-Being Study, which collected data on students’ mental health during the first wave of the COVID-19 pandemic in 26 countries, and multilevel modeling was applied.

**Results:**

It finds that, overall, female students reported more depressive feelings, and male students reported more excessive alcohol consumption. The strictness of the governmental containment measures explained a substantial amount of these gender differences in depressive feelings, but not in excessive alcohol consumption.

**Conclusions:**

Our study highlights that the COVID-19 pandemic had a gendered impact on students’ mental health. Studies that ignore the gendered impact of the COVID-19 pandemic are therefore limited in scope.

**Supplementary Information:**

The online version contains supplementary material available at 10.1186/s13690-023-01211-2.

**Table:** 

**Text box 1. Contributions to the literature**
- In the context of the COVID-19 pandemic, higher-education students have been identified as a risk group for mental health problems.
- Available research has predominantly focused on internalizing disorders, while externalizing disorders have been studied to a lesser degree.
- This study focuses on both depressive feelings and excessive alcohol consumption.
- It finds that, overall, female students reported more depressive feelings, and male students reported more excessive alcohol consumption.
- The strictness of the governmental containment measures explained a substantial amount of these gender differences in depressive feelings, but not in excessive alcohol consumption.

## Background

Mental health problems are a common phenomenon within higher-education students, but its manifestation differs between male and female students. While the latter group bears a greater risk of developing internalizing problems, with depression being particularly prevalent, these problems manifest themselves in male students mainly via externalizing disorders, with alcohol abuse being the most prevalent [1]. Moreover, male and female students also appear to differ in their vulnerability to the stressors connected to studying and student life and how they cope with these stressors [2, 3].

These gendered patterns in mental health vulnerabilities may have increased during the COVID-19 pandemic, as students were confronted with a range of containment measures, which had a substantial impact, both on their personal and academic life. Recent evidence indeed points to the devastating effect of these measures on young adults’ mental health [4, 5]. The restrictions on social life, such as the change to distance learning and minimized face-to-face encounters, resulted in a reduction in academic support and increased difficulties to focus during lectures [6–8]. Additionally, students reported higher financial and health-related worries due to economic stagnation and general COVID-19 outbreak respectively [9, 10]. Finally, female students have been found to report worse mental health outcomes in association with the pandemic than male students [11]. In countries with stricter and broader implementations of COVID-19 protective measures, higher levels of depressive feelings in students were also found [2]. However, the focus has been predominantly on internalizing disorders such as feelings of depression and anxiety burdening women in particular, while externalizing disorders, which particularly burden men, have been studied to a lesser degree. Available studies reveal contradicting patterns, with some studies finding a reduction in alcohol consumption in both male and female students [12], while other studies found an increase in alcohol consumption [13]. Studies focused on gendered patterns in alcohol consumption during the COVID-19 pandemic found that men reported substantially higher levels of alcohol abuse compared to female students [14–16].

The aim is twofold: (1) to examine the extent to which gender patterns in both indicators of mental health vary by the stringency of the COVID-19 measures implemented by the government; and (2) to examine the extent to which these associations are mediated by stressors related to student life and/or to newly arising stressors in the context of the first wave of the COVID-19 pandemic.

## Methods

### Data

The COVID-19 International Student Well-being Study (C19 ISWS) is used, which collected information on student well-being through an online survey during the first wave of the COVID-19 pandemic in 133 HEIs in 26 countries. Except South Africa, Israël and Russia, all included countries are located in Europe. Details about the study procedures can be found in the study protocol [17]. For this study, a subsample of the data was used to cover each participating country during a period with relatively stable policy measures (see Tables A1 and Table A2 in the Appendix). A random selection of 1,000 cases was drawn in countries with a larger size sample in order to correct for an overrepresentation of these countries in the total sample. This resulted in an analytical sample consisting of 20,103 respondents (73,9% is female, 78,3% is below the age of 26). Descriptive statistics are presented in Table A3.

### Measures

An eight-item version of the Center for Epidemiologic Studies–Depression Scale (CES-D-8) scale was used to measure the frequency and severity of depressive feelings [18, 19]. Respondents were asked to indicate how often in the week previous to the survey they felt or behaved in a certain way (felt depressed, felt that everything was an effort, slept poorly, felt lonely, felt sad, could not get going, enjoyed life, or felt happy – last two items are reverse-coded). Response categories forming a 4-point Likert scale ranged from *none* or *almost none of the time* (0) to *all* or *almost all of the time* (3). Scale scores were assessed using a non-weighted summed rating and ranged from 0 to 24, with higher scores indicating a higher frequency and severity of depressive feelings. In the C19 ISWS sample, the country-specific Cronbach’s alphas ranged between 0.85 and 0.90 [17].

A measure of *heavy episodic drinking* (HED), defined in line with the definition of the WHO (2022) was used. HED was defined as drinking at least 60 grams of pure alcohol on one single occasion in the last seven days. Students reporting that they ‘never’ or ‘less than once a week’ drank six glasses of alcohol (as an approximation of 60 grams of pure alcohol) on a single occasion scored ‘0’ on the dichotomous variable HED and those answering ‘once a week’, ‘more than once a week’ or ‘(almost) daily’ scored ‘1’.

In addition to *gender*, *age*, *migrant background*, *parental level of education* and *study program* were taken into account. Age was included as a dichotomous variable to avoid a strong overlap with study program, which distinguished first-year students from other students. The highest level of education attained by either parent was used as a proxy of their socioeconomic position [20].

In order to assess the level of *academic stress* during the COVID-19 pandemic, respondents were asked to what degree they agreed with the statements: (1) My university/college workload has significantly increased since the COVID-19 outbreak; (2) I know less about what is expected of me in the different course modules/units since the COVID19 outbreak; (3) I am concerned that I will not be able to successfully complete the academic year due to the COVID-19 outbreak; and (4) The change in teaching methods resulting from the COVID − 19 outbreak has caused me significant stress. Each item was rated on a 5-point Likert scale ranging from 0 (strongly disagree) to 4 (strongly agree). The scale had a Cronbach’s alpha 0,7 and country-specific reliability indices are reported elsewhere (17). *Worries to get (re)infected with COVID-19* was measured by a scale ranging from 0 (no worries) to 10 (very worried about getting (re)infected by COVID-19). *Change in financial situation* was based on the difference in answers to the statement ‘I had sufficient financial resources to cover my monthly costs’, thereby comparing their situation before the COVID-19 outbreak with that of their situation during the week prior to filling out this survey. The variable consists of four categories: (1) no change: not struggling, (2) no change: struggling with financial resources, (3) worse than before the COVID-19 outbreak, and (4) better than before the COVID-19 outbreak. Finally, *participation in social activities* was included as a categorical variable differentiating between students who had (1) online and face to face (FTF) social activities (= ref), (2) no social activities, (3) only FTF, and (4) only online activities.

To assess the *stringency of COVID-19 protective measures* implemented in each country, we used national measures of the University of Oxford coronavirus government response tracker (OxCGRT) stringency index, and regional measure for New Jersey and Quebec [20]. To take the strength and timing (in relation to the survey period) of the pandemic into account, we calculated the *country’s level of excess mortality* (p-score) during the period of the data collection, using data from Eurostat (2020) or national or regional statistics bureaus, and additionally used this variable to calculate *the timing of the survey in relation to the peak* of the first wave of the COVID-19 pandemic with three categories (0) before the peak, (1) during and (2) after the peak of the first wave of the COVID-outbreak. In addition, we included the control variable real *GDP growth rate* 2020 as an indicator of the socioeconomic condition of the country. An overview of these macrolevel indicators is provided in Appendix Table A4.

### Statistical analyses

A hierarchical three-level model was constructed which clustered respondents within HEIs (N = 125), which were again clustered within countries (N = 26). Multilevel linear and logistic regression analyses were performed with respectively depressive feelings and HED as dependent variables, and with a random slope for gender included. In the first model, gender differences were estimated, controlled for the relevant individual and country level control variables. In the second model, a cross-level interaction between gender and the stringency index was included to estimate whether the gender differences in depression and HED varied by the stringency of the COVID-19 measures. In a third model, the individual stressors were added to examine whether they mediated the relation between gender, the stringency index, and depression or HED.

Sensitivity analyses were performed with the number of glasses of alcohol (e.g., a glass of wine, a shot, or a glass of beer between 25 and 33 cl) on average per week during the COVID-19 outbreak as dependent variable. As this is a count variable, negative binomial models were estimated (Table A5 in the appendix).

Data preparation and descriptive statistics were done in SPSS® version 26, and the multilevel analyses were performed in MLwiN Version 3.05.

## Results

Results in Table 1, Model 1 again show that female students reported significantly more depressive feelings compared to male students (B = 0.699; p < 0.001), controlled for socio-demographic, socioeconomic, academic factors and country-level control variables. In countries with stricter containment measures, students reported slightly more depressive feelings (B = 0.056; p < 0.05). As Model 2 shows, the effect of living in a country with stricter stringency measures was significantly more pronounced in female compared to male students (B = 0.018; p < 0.05).

**Table 1:** Multilevel linear regression analyses results for gender differences in depressive feelings, controlling for the national level of stringency, for socio-demographic, socioeconomic, and academic factors, for COVID-19 related factors, and for relevant country-level control variables

		Model 1			Model 2			Model 3		
		B	S.E.		B	S.E.		B	S.E.	
**Gender** (ref. men)										
Women		0.699	0.098	***	0.697	0.087	***	0.279	0.085	***
**Stringency index**		0.056	0.024	*	0.047	0.025	a	0.006	0.020	
Gender * Stringency index					0.018	0.008	*	0.012	0.008	
**Individual-level factors**										
**Socio-demographic, socioeconomic, and academic factors**										
	**Age** (ref. 17–25)									
	>=26	-0.557	0.093	***	-0.555	0.093	***	-0.261	0.085	**
	**Migration status** (ref. no)									
	First generation migrant background	0.646	0.105	***	0.646	0.105	***	0.264	0.096	**
	Second generation migrant background	0.603	0.118	***	0.606	0.118	***	0.436	0.107	***
	**Education parents** (ref. High)									
	Low educational background	0.406	0.136	**	0.406	0.136	**	-0.015	0.124	
	Moderate educational background	0.248	0.082	**	0.248	0.082	**	-0.069	0.075	
	**Study program** (ref. first-year bachelor)									
	Bachelor (not in the first year)	0.218	0.094	*	0.217	0.094	*	-0.019	0.085	
	Master program	-0.298	0.091	***	-0.300	0.091	***	0.060	0.083	
	Other programs	-0.505	0.236	*	-0.504	0.236	*	-0.599	0.214	**
**COVID-19 related factors**										
	**Worries to get infected with COVID-19**							0.198	0.011	***
	**Academic stress**							0.463	0.009	***
	**Participation in social activities** (ref. both online and face to face)									
	No social activities							1.199	0.214	***
	Only face to face							0.109	0.181	
	Only online							0.759	0.075	***
	**Financial situation** (ref. no change: not struggling)									
	No change: struggling with financial resources							1.550	0.170	***
	Change: worse during covid							1.203	0.075	***
	Change: better during covid							0.558	0.126	***
**Country-level control variables**										
	**Timing survey** (ref. After COVID-19 peak)									
	Before the COVID-19 peak	0.335	0.62		0.323	0.631		0.217	0.495	
	During the COVID-19 peak	-0.312	0.646		-0.259	0.657		-0.457	0.517	
	**Real GDP growth rate**	-0.335	0.208		-0.352	0.212		-0.140	0.166	
	**Excess mortality rate**	-1.842	2052		-1.907	2083		-0.955	1645	
**Variance**										
	Country level	0.863	0.322		0.920	0.335		0.539	0.207	
	Random slope gender	0.074	0.063		0.028	0.048		0.043	0.045	
	HEI level	0.582	0.138		0.582	0.138		0.376	0.095	
	Individual-level	23.982	0.24		23.982	0.240		19.805	0.199	
	-2LL	120692.2			120688.1			116837.6		

* p < 0,05; **p < 0,01; *** p < 0,001

Model 3 suggests that the COVID-19-related stressors mediate the association between gender and depressive feelings, as the effect of gender reduced by more than half after taking these stressors into account (B_gender_=0.279, p < 0.001 and b_gender*stringency_ =0.012, p > 0.05). While students who were more worried to get (re-)infected with COVID-19 (B = 1.198, p < 0.001), and students with elevated levels of academic stress (B = 0.463, p < 0.001), and students who experienced an increase in financial worries during the pandemic (B = 1.203, p < 0.001) also reported more depressive feelings, the bivariate results (Appendix Table A3) show that these stressors are more prevalent among female than male students. Students who did not participate in any social activities (B = 1.199, p < 0.001), as well as students who only participated in online activities (B = 0.759; p < 0.001) reported more depressive feelings, compared to students who participated in both online and offline social activities. Model 3 additionally revealed that the significant interaction between gender and the stringency index can be largely attributed to these COVID-19 related stressors, as the interaction effect between gender and the stringency index was no longer significant.

The same models were estimated for the dependent binary variable to measure excessive alcohol consumption. Results in Table 2, Model 1 reveal that male students were significantly more likely to report excessive alcohol consumption (OR = 0.554, p < 0.001) compared to female students. The odds of excessive alcohol consumption were however not lower in countries with stricter containment measures (OR = 1.280, p > 0.05). Model 2 revealed that the effect of the stringency index did not differ between male and female students in a significant manner either.

**Table 2:** Multilevel logistic regression analyses results for gender differences in excessive alcohol consumption, controlling for the national level of stringency, for socio-demographic, socioeconomic, and academic factors, for COVID-19 related factors, and for relevant country-level control variables

			Model 1			Model 2				Model 3				
			OR	Sign.		OR		Sign.		OR		Sign.		
**Gender** (ref. men)														
Women			0.554	***		0.546		***		0.543		***		
**Stringency index**			1.280			1.292				1.480				
Gender * stringency index						0.993				0.993				
**Individual-level factors**														
	**Socio-demographic. socioeconomic. and academic factors**													
		**Age** (ref. 17–25)												
		>=26	1.066			1.065				1.133				
		**Migration status** (ref. no)												
		First-generation migrant background	0.870			0.869				0.873				
		s-generation migrant background	0.714	***		0.713		***		0.719		***		
		**Education parents** (ref. High)												
		Low educational background	0.823			0.822				0.791		*		
		Moderate educational background	0.981			0.981				0.948				
		**Study program** (ref. first-year bachelor)												
		Bachelor or Master’s program (not in the first year)	0.909			0.910				0.919				
		Other programs	0.853			0.851				0.828				
	**COVID-19 related factors**													
		**Worries to get infected with COVID-19**								0.950		***		
		**Academic stress**								1.047		***		
		**Participation in social activities** (ref. both online and face to face)												
		No social activities							1.008					
		Only face to face								1.176				
		Only online								0.670		***		
		**Financial situation** (ref. no change: not struggling)												
		No change: struggling with financial resources							1.092					
		Change: worse during covid							1.483		***			
		Change: better during covid								1.301		**		
	**Country-level control variables**													
		**Timing survey** (ref. After COVID-19 peak)												
		Before the COVID-19 peak	0.926			0.927				0.913				
		During the COVID-19 peak	1.380			1.374				1.394				
		**Real GDP growth rate**	0.989			0.989				0.991				
		**Excess mortality rate**	0.976	***		0.980		*		0.979		**		
	**Variance**													
		Country-level	0.058	0.031		0.058		0.031		0.054		0.029		
		Random slope gender	0.000	0.000		0.000		0.000		0.000		0.000		
		HEI level	0.061	0.027		0.062		0.027		0.052		0.025		

* p < 0,05; **p < 0,01; *** p < 0,001

In Model 3 various COVID-19-related factors were added. The more worried students were to get (re-)infected with COVID-19, the lower their odds of excessive alcohol consumption (B = 0.950, p < 0.001). In the same line, students who only participated in online social activities were less likely to report excessive alcohol consumption (B = 0.670, p < 0.001), compared to students who participated in both online and face to face activities. Excessive alcohol consumption was also positively related to higher levels of academic stress (OR = 1.047, p < 0.001). A change in the financial situation acted as both as a deteriorator (OR = 1.483, p < 0.001) and as an improvement (OR = 1.301, p < 0.01), as both groups were more likely to report excessive alcohol consumption compared to students who experiences no change in their financial situation. Contrary to the results for depressive feelings, the inclusion of these COVID-19-related stressors in the model did not weaken the gender effect. Male students were still significantly more likely to report excessive alcohol consumption (OR = 0.543, p < 0.001) compared to female students, even when taking all the other factors into account.

The additional sensitivity analysis with the average number of glasses of alcohol per week overall reveals the same patterns: alcohol consumption was significantly lower in students with a migrant background compared to students without a migrant background, and lower in students who were more worried to get infected with COVID-19, who had lower levels of academic stress, and who only had online social interactions. However, the number of drinks students had was also significantly lower in countries with stricter containment measures, but a significant gender difference therein could not be established either.

## Discussion

Our cross-national study finds that the level of depressive feelings was higher in countries with stricter containment measures, which confirms the available literature [4] and that overall female students reported significantly more depressive symptoms than male students within that context. This suggests that the secondary effects of these types of measures seem to affect female students in particular. In our analysis, this gendered effect of the containment measures was fully explained by COVID-19 related stressors, such as the degree to which students were worried to get infected with COVID-19, their level of academic stress that was caused by the pandemic, a reduction in face-to-face social interactions, and changes in the financial situation of these students during the pandemic. Each of these stressors was related to higher levels of depressive feelings, and fully explained the interaction between gender and the stringency index. These results suggest improvements can be made to governmental reactions and measurements considering a potential new pandemic in the future.

At the same time, excessive alcohol consumption seemed not to be related to the stringency index, but rather by individual level stressors, regardless of the context. A higher risk of excessive alcohol consumption was found in students who reported higher levels of academic stress, and was related to changes in the financial situation of these students during the pandemic. In these cases, excessive alcohol consumption may have been applied as a coping resource against distress. A lower risk of excessive alcohol consumption was found in students who were more worried to get infected with COVID-19, as well as among students who only have online social interactions. Alcohol consumption is associated with social activities in many countries, much of which fell away during the pandemic, explaining these findings. Some studies point out that consumption in alcohol, particularly within the context of the pandemic, was more likely to increase in older age groups compared to those aged 25 and under. Most studies even reported decreases in alcohol consumption among emerging adults, an effect that was particularly pronounced among college students [21]. A UK based study found a substantial decrease in binge drinking during the first lock-down period as well [22]. While our study finds that female students are less likely to have used excessive alcohol during the first wave of the COVID-19 pandemic compared to male students, this gender difference was not related to the stringency of the containment measures either.

It is striking, however, that even though our overall sample found gender differences in both depressive feelings and alcohol consumption, when looking at specific countries, such differences were only detected in a small proportion of countries. in about half of the observed countries, male and female students reported similar patterns of depressive symptoms and alcohol consumption, which may reflect a substantial increase in psychological distress in both female and male students, something which has been confirmed by longitudinal studies [22]. In the same line, we observed that in the countries where the most pronounced gender differences were noted, remarkably high levels of depressive symptoms were also found in both groups. This seems to suggest that a substantial portion of the distress resulting from the pandemic does not appear to be fully gendered, but has a similar effect on both genders.

The available single-country literature did not establish consistent gender differences in pandemic-related changes in depressive feelings and alcohol consumption either. Related to depressive feelings, most find a substantial increase in depressive feelings [23, 24]. However, while some studies find that this increase was more pronounced in females than males [25, 26], other research finds more elevated levels of depression in males compared to females [27]. The cross-national variation found in our study highlights that single-country data cannot be extrapolated to other contexts, particularly within the context of the COVID-19 pandemic.

Turning to alcohol consumption, the available literature comes to contradicting conclusions [28], or identified gender differences in drinking contexts. For example, the study by Dumas and colleagues [29] finds greater increases in females in overall drinking, and in drinking with parents, whereas males were more likely to report both solitary drinking and in-person drinking with peers. Another study found that solitary drinking increased among adult men, although overall consumption levels generally did not differ by gender [30]. Further research is needed to examine how these drinking contexts may explain our cross-national variation in excessive alcohol consumption among male and female students within the context of the COVID-19 pandemic.

Our study has several limitations. First, because we made use of cross-sectional data, we were unable to disentangle causal paths between depressive symptoms, excessive alcohol consumption and the various social stressors, nor were we able to examine the degree to which both outcomes changed since the first wave of the COVID-19 pandemic. One could also assume mental health, including symptoms of depression and alcohol abuse, may have deteriorated in the second and third COVID waves. Second, while the entire student population was asked to participate in most of the HEIs that participated in the C19 ISWS, the sample remains non-representative., both in terms of the selection of students, HEIs, and countries. This also brings along the issue of self-selection into the C19 ISWS; it may be likely that students who experienced stress due to the COVID-19 pandemic were also more likely to participate in a study that focusses on this topic. Students with a more disadvantaged socioeconomic background or limited access to the internet are generally less likely to participate in surveyswhile female students are more likely to do so, to which the C19 ISWS is also subject. Hence, the disproportionate representation of female compared to male participants in this study may have influenced the overall prevalence rates of our outcome variables. Further, while respondents had the opportunity to identify as the gender category X, this subpopulation was removed from the sample because of too few respondents within this category. Finally, this study only included high-income and upper-middle-income countries. Further research focused on countries with less developed economies and/or differential COVID-19 measures might result in different outcomes. eaders should keep these limitations in mind when interpreting our results.

## Conclusion

This study is the first to explore the gender difference in depressive feelings and excessive alcohol consumption in the context of the COVID-19 pandemic in a wide variety of countries. It shows that the COVID-19 pandemic was uniquely translated into both mental health problems. It found that depressive feelings were higher in countries with stricter containment measures, particularly in female students. The study also confirmed that male students were more likely to report excessive alcohol consumption than female students, but this difference did not appear to be explained by the COVID-19 protective measures implemented during the first wave of the COVID-19 pandemic.

### Electronic supplementary material

Below is the link to the electronic supplementary material.

**Appendix: Table A1**. Selection of survey period per country. **Table A2**: Overview of the selected survey periods per country. **Table A3**: Descriptive statistics and bivariate statistics of depressive feelings and excessive alcohol consumption as well as all level-1 explanatory and control variables by gender. **Table A4**: Macro-level variables by country. **Table A5**: Results of the negative binomial multilevel analysis with the average number of glasses of alcohol during the first wave of the COVID-19 pandemic.

## Data Availability

The rough data and analytical sample underlying this article are available in the public domain: https://zenodo.org/search?page=1&size=20&q=C19%20ISWS.
