# Supplementary material for: An international comparison of gender differences in mental health among higher-education students during the first wave of the COVID-19 pandemic: a multilevel design
Source: Arch Public Health. 2023 Dec 7;81:211. doi: 10.1186/s13690-023-01211-2 (PMC10701939; doi:10.1186/s13690-023-01211-2)
Supplement: Supplementary file 1 — Appendix: Table A1. Selection of survey period per country. Table A2: Overview of the selected survey periods per country. Table A3: Descriptive statistics and bivariate statistics of depressive feelings and excessive alcohol consumption as well as all level-1 explanatory and control variables by gender. Table A4: Macro-level variables by country. Table A5: Results of the negative binomial multilevel analysis with the average number of glasses of alcohol during the first wave of the COVID-19 pandemic. [file 13690_2023_1211_MOESM1_ESM.docx]

APPENDIX

**Table A1. Selection of survey period per country**

| There was a temporal variation of the stringency index within countries' periods of data collection, but this variation was not enough for the inclusion of an additional level 'country*period' in the multilevel analyses to model periodic changes in the stringency index. As a result, we decided to limit the survey period per country to cover only the period with relatively stable policy measures. Only respondents who participated in the survey during this selected survey period were included. In some countries, all the collected data was used (Czech, Iceland, South Africa, USA). For most countries, the survey period was shortened by one to four weeks (Belgium (one week excluded), Canada (1), Cyprus (2), Denmark (2), Finland (1), France (5), Germany (4), Greece (3), Israel (2), Italy (3), Portugal (1), Romania (4), Slovakia (2), Norway (2), Spain (4), Switzerland (3), Turkey (1), and UK (2). If the basic rule could not be applied because the first two weeks were not stable in terms of policy measures, we selected a period in between. This was the case for Hungary, the Netherlands, and the UK. The selection of the sample period resulted in some countries in the exclusion of an HEI, as these institutions had launched their survey at a different time period than the other participating HEIs within that country. |
| --- |

**Table A2: Overview of the selected survey periods per country**

| **Week** | 12 | 13 | 14 | 15 | 16 | 17 | 18 | 19 | 20 | 21 | 22 | 23 | 24 | 25 | 26 | 27 | 28 | 29 | 30 | 31 |
| --- | --- | --- | --- | --- | --- | --- | --- | --- | --- | --- | --- | --- | --- | --- | --- | --- | --- | --- | --- | --- |
| **Date** |  |  |  |  |  |  | 27/04-03/05 | 04/05-10/05 | 11/05-17/05 | 18/05-24/05 | 25/05- 31/05 | 01/06-07/06 | 08/06-14/06 | 15/06-21/06 | 22/06-28/06 | 29/06-  05/07 | 06/07-12/07 | 13/07-19/07 | 20/07-26/07 | 24/07-02/08 |
| Belgium |  |  |  | **P** |  |  |  |  |  |  |  |  |  |  |  |  |  |  |  |  |
| Canada |  |  |  |  |  |  | **P** |  |  |  |  |  |  |  |  |  |  |  |  |  |
| Czech Republic |  |  |  |  |  |  |  |  |  |  |  |  |  |  |  | **P** |  |  |  |  |
| Cyprus |  |  | **p** |  |  |  |  |  |  |  |  |  |  |  |  |  |  |  |  |  |
| Denmark |  |  | **P** |  |  |  |  |  |  |  |  |  |  |  |  |  |  |  |  |  |
| Finland |  |  |  | **P** |  |  |  |  |  |  |  |  |  |  |  |  |  |  |  |  |
| France |  |  |  |  |  |  |  |  | **P** |  |  |  |  |  |  |  |  |  |  |  |
| Germany |  |  | **P** |  |  |  |  |  |  |  |  |  |  |  |  |  |  |  |  |  |
| Greece |  |  |  | **P** |  |  |  |  |  |  |  |  |  |  |  |  |  |  |  |  |
| Hungary |  | **P** |  |  |  |  |  |  |  |  |  |  |  |  |  |  |  |  |  |  |
| Iceland |  |  |  | **P** |  |  |  |  |  |  |  |  |  |  |  |  |  |  |  |  |
| Israel |  |  |  |  |  |  |  | **P** |  |  |  |  |  |  |  |  |  |  |  |  |
| Italy |  |  | **P** |  |  |  |  |  |  |  |  |  |  |  |  |  |  |  |  |  |
| The Netherlands |  | **P** |  |  |  |  |  |  |  |  |  |  |  |  |  |  |  |  |  |  |
| Norway |  |  |  | **P** |  |  |  |  |  |  |  |  |  |  |  |  |  |  |  |  |
| Portugal |  | **P** |  |  |  |  |  |  |  |  |  |  |  |  |  |  |  |  |  |  |
| Romania |  |  |  |  |  |  |  |  |  |  |  |  |  |  |  | **P** |  |  |  |  |
| Russia |  |  |  |  |  |  |  | **P** |  |  |  |  |  |  |  |  |  |  |  |  |
| Slovakia |  |  |  |  |  |  |  |  |  |  |  |  | **P** |  |  |  |  |  |  |  |
| South Africa |  |  |  |  |  |  |  |  |  |  |  |  |  |  |  |  |  | **P** |  |  |
| Spain |  |  |  |  |  | **P** |  |  |  |  |  |  |  |  |  |  |  |  |  |  |
| Sweden |  |  |  |  | **p** |  |  |  |  |  |  |  |  |  |  |  |  |  |  |  |
| Switzerland |  |  |  |  | **P** |  |  |  |  |  |  |  |  |  |  |  |  |  |  |  |
| Turkey |  |  | **P** |  |  |  |  |  |  |  |  |  |  |  |  |  |  |  |  |  |
| UK | **P** |  |  |  |  |  |  |  |  |  |  |  |  |  |  |  |  |  |  |  |
| USA |  |  |  |  | **P** |  |  |  |  |  |  |  |  |  |  |  |  |  |  |  |

Legend: Green = selected survey period; Red = unselected and excluded from the survey period; P = peak of first COVID-19 wave based on excess mortality rate

**TABLE A3:** Descriptive statistics and bivariate statistics of depressive feelings and excessive alcohol consumption as well as all level-1 explanatory and control variables by gender.

|  | **Total** | | **Men** | **Women** | **Depressive feelings** | | | **Excessive alcohol consumption** | |
| --- | --- | --- | --- | --- | --- | --- | --- | --- | --- |
|  | **N** | **%** | **%** | **%** | **x** | **sd** | **sig.^a^** | **%** | **sig.^b^** |
| **Gender** |  |  |  |  |  |  | *** |  | *** |
| Men | 5215 |  |  |  | 9.3 | 5.2 |  | 19.2 |  |
| Women | 14817 |  |  |  | 10.0 | 5.1 |  | 11.5 |  |
| **Age** |  |  |  |  |  |  | *** |  | *** |
| 17-25 | 15685 | 78.3 | 75.5 | 79.3 | 10.1 | 5.2 |  | 14.8 |  |
| 26 or older | 4347 | 21.7 | 24.5 | 20.7 | 8.9 | 4.9 |  | 8.8 |  |
| **Migrant background** |  |  |  |  |  |  | *** |  | *** |
| No | 14957 | 74.7 | 71.8 | 75.7 | 9.8 | 5.1 |  | 14.0 |  |
| 1ste generation migrant background | 2959 | 14.8 | 17.8 | 13.7 | 10.2 | 5.1 |  | 11.0 |  |
| 2nd generation migrant background | 2116 | 10.6 | 10.4 | 10.6 | 10.1 | 5.1 |  | 13.2 |  |
| **Education parents** |  |  |  |  |  |  | *** |  | *** |
| Low educational background | 1688 | 8.4 | 8.1 | 8.6 | 10.3 | 5.4 |  | 10.2 |  |
| Moderate educational background | 5695 | 28.4 | 26.8 | 29.0 | 10.1 | 5.2 |  | 12.4 |  |
| High educational background | 12649 | 63.1 | 65.1 | 62.5 | 9.7 | 5.1 |  | 14.4 |  |
| **Study program** |  |  |  |  |  |  | *** |  | *** |
| First year bachelor | 10869 | 54.3 | 53.0 | 54.7 | 10.0 | 5.1 |  | 13.9 |  |
| Bachelor program (not in the first year) | 8690 | 43.4 | 44.6 | 43.0 | 9.7 | 5.1 |  | 13.3 |  |
| Other program | 473 | 2.4 | 2.4 | 2.3 | 9.2 | 5.2 |  | 7.2 |  |
| **Participation in social activities** |  |  |  |  |  |  | *** |  | *** |
| No social activities | 458 | 2.3 | 3.1 | 2.0 | 11.2 | 6.2 |  | 8.7 |  |
| Only face to face | 645 | 3.2 | 4.4 | 2.8 | 9.9 | 5.4 |  | 15.1 |  |
| Only online | 5193 | 25.9 | 26.8 | 25.6 | 10.8 | 5.3 |  | 9.2 |  |
| Both online and face to face | 13736 | 68.6 | 65.6 | 69.6 | 9.5 | 4.9 |  | 15.2 |  |
| **Financial situation** |  |  |  |  |  |  | *** |  | *** |
| No change: not struggling with financial resources | 12162 | 60.7 | 63.7 | 59.7 | 8.9 | 4.9 |  | 12.8 |  |
| No change: struggling with financial resources | 765 | 3.8 | 3.6 | 3.9 | 12.0 | 5.5 |  | 11.9 |  |
| Change: worse during covid | 5693 | 28.4 | 25.3 | 29.5 | 11.4 | 5.1 |  | 14.2 |  |
| Change: better during Covid | 1412 | 7.0 | 7.5% | 6.9 | 10.1 | 5.1 |  | 16.8 |  |
|  | **N** | **x(sd)** | **x(sd)** | **x(sd)** | **R** | **sig.^c^** | **binge drinking: x(sd)** | **no binge drinking: x(sd)** | **sig.^a^** |
| **Worries to get infected with COVID-19** (0-10) | 19037 | 3.7(3.2) | 3.0(2.9) | 4.0(3.2) | 0.198 | *** | 3.5(3.2) | 3.8(3.2) | *** |
| **Academic stress** (0-16) | 20032 | 9.5(3.9) | 9.1(3.9) | 9.6(3.8) | 0.410 | *** | 9.8(3.9) | 9.4(3.8) | *** |
| *a Anova test, b Chi², c Pearson's correlation* | | | | | | | | | |

**Table A4: Macro-level variables by country**

| **Country** | **Stringency index (0-100)** | **Real GDP Growth Rate** | **Excess Mortality Rate** | | **Timing Peak** | |
| --- | --- | --- | --- | --- | --- | --- |
| Belgium | 81,48 | 1,40 | 0,20 | After peak | |  |
| Canada | 78,70 | 1,70 | 0,40 | After peak | |  |
| Czech Rep. | 57,41 | 2,50 | -0,03 | During peak | |  |
| Denmark | 68,52 | 2,40 | -0,01 | After peak | |  |
| Finland | 56,48 | 0,90 | 0,05 | After peak | |  |
| France | 76,85 | 1,50 | 0,14 | During peak | |  |
| Germany | 59,98 | 0,60 | 0,00 | After peak | |  |
| Greece | 68,52 | 1,90 | 0,02 | After peak | |  |
| Hungary | 66,67 | 4,90 | -0,06 | After peak | |  |
| Iceland | 39,81 | 1,90 | -0,03 | After peak | |  |
| Israel | 84,53 | 3,50 | 0,17 | During peak | |  |
| Italy | 63,89 | 0,30 | 0,02 | After peak | |  |
| Netherlands | 71,30 | 1,80 | -0,02 | After peak | |  |
| Norway | 45,64 | 1,20 | 0,03 | After peak | |  |
| Portugal | 75,00 | 2,20 | 0,19 | After peak | |  |
| Romania | 87,04 | 4,10 | 0,06 | During peak | |  |
| Russia | 85,19 | 1,40 | 0,31 | During peak | |  |
| Slovakia | 75,00 | 2,40 | -0,02 | After peak | |  |
| South Africa | 76,85 | 0,20 | 0,14 | Before peak | |  |
| Spain | 77,58 | 2,00 | -0,04 | After peak | |  |
| Sweden | 59,26 | 1,20 | -0,03 | After peak | |  |
| Switzerland | 69,44 | 1,00 | 0,04 | After peak | |  |
| Turkey | 75,93 | 0,90 | 0,04 | After peak | |  |
| UK | 61,11 | 1,40 | 0,02 | After peak | |  |
| USA | 78,70 | 2,30 | 0,48 | After peak | |  |
| Cyprus | 76,85 | 3,30 | 0,02 | After peak | |  |

**Table A5: Results of the negative binomial multilevel analysis with the average number of glasses of alcohol during the first wave of the COVID-19 pandemic**

|  |  |  | **Model 1** | | **Model 2** | | **Model 3** | |
| --- | --- | --- | --- | --- | --- | --- | --- | --- |
|  |  |  | OR | Sign. | OR | Sign. | OR | Sign. |
| **Gender** (ref. men) | | |  |  |  |  |  |  |
| Women | | 0.678 | *** | 0.68 | *** | 0.678 | *** |  |
| **Stringency index** | | | 0.987 | * | 0.984 | * | 0.985 | * |
| **Gender * stringency index** | | |  |  | 1.004 |  | 1.003 |  |
|  | | |  |  |  |  |  |  |
| **Individual-level factors** | | |  |  |  |  |  |  |
|  | **Socio-demographic. socioeconomic. and academic factors** | |  |  |  |  |  |  |
|  | **Age** (ref. 17-25) |  |  |  |  |  |  |  |
|  | >=26 | 1.162 | ** | 1.162 | ** | 1.208 | *** |  |
|  |  | **Migration status** (ref. no) |  |  |  |  |  |  |
|  | First-generation migrant background | 0.845 | ** | 0.845 | ** | 0.856 | ** |  |
|  | Second-generation migrant background | 0.725 | *** | 0.725 | *** | 0.735 | *** |  |
|  |  | **Education parents** (ref. High) |  |  |  |  |  |  |
|  | Low educational background | 0.823 | *** | 0.654 | *** | 0.628 | *** |  |
|  | Moderate educational background | 0.981 |  | 0.927 |  | 0.907 | * |  |
|  |  | **Study program** (ref. first-year bachelor) |  |  |  |  |  |  |
|  |  | Bachelor or Master’s program (not in the first year) | 0.964 |  | 0.963 |  | 0.985 |  |
|  |  | Other programs | 0.787 |  | 0.787 |  | 0.795 |  |
|  | **COVID-19 related factors** | |  |  |  |  |  |  |
|  |  | **Worries to get infected with COVID-19** |  |  |  |  | 0.964 | *** |
|  |  | **Academic stress** |  |  |  |  | 1.013 | * |
|  |  | **Participation in social activities** (ref. both online and face to face) |  |  |  |  |  |  |
|  |  | No social activities |  |  |  |  | 0.665 | ** |
|  |  | Only face to face |  |  |  |  | 0.960 |  |
|  |  | Only online |  |  |  |  | 0.657 | *** |
|  |  | **Financial situation** (ref. no change: not struggling) |  |  |  |  |  |  |
|  |  | No change: struggling with financial resources |  |  |  |  | 0.928 |  |
|  |  | Change: worse during covid |  |  |  |  | 1.235 | *** |
|  | Change: better during covid |  |  |  |  | 1.320 | *** |  |
| **Country-level control variables** | |  |  |  |  |  |  |  |
|  | **Timing survey** (ref. After COVID-19 peak) |  |  |  |  |  |  |  |
|  | Before the COVID-19 peak | 0,970 |  | 0.966 |  | 0.973 |  |  |
|  | During the COVID-19 peak | 0,943 |  | 0.943 |  | 0.990 |  |  |
|  |  | **Real GDP growth rate** | 0.983 |  | 0.983 |  | 0.991 |  |
|  |  | **Excess mortality rate** | 1.078 |  | 1.073 |  | 1.153 |  |
|  | **Variance** | |  |  |  |  |  |  |
|  | Country-level | 0,029 | 0.024 | 0.031 | 0.024 | 0.001 | 0.001 |  |
|  | Random slope gender | 0,004 | 0.011 | 0.004 | 0.011 | 0.001 | 0.009 |  |
|  | HEI level | 0,116 | 0.029 | 0.112 | 0.029 | 0.164 | 0.033 |  |
